# The Influence of Climate in Chronic Diseases

**Published:** 1830-07-01

**Authors:** 


### THE INFLUENCE OF CLIMATE IN CHRONIC DISEASES, &c. By *James Clark*, M. D. Second Edition. 1830.

IN a former number we gave an ample analysis of Dr. Clark's valuable work, and we are happy to see a new edition so soon in the field. In this article we shall only notice, of course, the additions which Dr. C. has made to the first edition. It appears that our indefatigable author took great pains, not only to collect information respecting the climate of various places to which invalids repair in this country, but to visit many of them personally, in order to judge for himself. The principal additions to the climate of England are under the heads of Undercliff in the Isle of Wight, and Clifton. The salubrious climate of the former is, however, rendered almost nugatory by the absence of accommodations for invalids. It is to be hoped that, in these days of speculation, some of our moneyed men will employ their capital in erecting houses along that romantic range of terraces on the South coast of the Wight, where more health might be secured than in any part of the boasted Italian territory. In this article, however, we must dedicate the few pages we have to spare, to a consideration of those localities which are inclosed within, or placed beyond the Atlantic Ocean.

There are various islands, or groups of islands, in the Northern Atlantic which deserve more consideration in respect to climate, than they have hitherto received. The Bahamas, Bermudas, Canaries, and Azores are the principal places worthy of notice.

I. The Bahama Islands are on the very borders of the Tropics; but their immediate vicinity to the American continent so modifies their climate, as to give it a very different character from that of the inter-tropical inlands. These islands are all low, and chiefly composed of a coarse sand and stone. They contain no natural springs, and the trade winds are not very regular there. The southernly winds are hot and oppressive, accompanied by a heavy deposition of dews in the night. The North wind frequently prevails and lowers the temperature. While the Winter temperature is nearly six degrees, and the Spring two degrees colder than Barbadoes, the Summer is three degrees hotter.

"The Bahama islands, generally speaking, are not unhealthy; although there is a considerable difference in this respect, between the different islands. That of New Providence, in which is the capital, Nassau, the only town in the colony, is not by any means one of the healthiest; on account chiefly of some swampy ground which it contains. The small island, called Harbour Island, close to Eleuthera, one of the largest of the group, is esteemed particularly healthy, and forms the chief resort of invalids and convalescents from New Providence. There are several other healthy spots, as on the island of Abaco; but at all these places there is a great deficiency of accommodations, and moreover, they are sixty miles distant from Nassau, the only place where medical advice is to be found." 4.

II. BERMUDAS.

This group consists of a numerous cluster of small islands, resembling in features and structure the Bahamas. The highest ground on any of them does not exceed 200 feet above the sea. The soil is arid, from the absorbing nature of the Bermuda rock, and there is hardly any other than rain water.

"The cool season, that is, from October till May, is the most healthy, and the only part of the year during which this climate is at all suited to invalids. One of the principal objections to Bermuda, as a winter residence for pulmonary invalids, is the prevalence of strong winds; which are such as still justify the epithet applied by Shakespeare to these islands, 'the still-vex'd Bermoothes.'

Of these winds the damp, oppressive south-west is the prevailing; but the most violent and injurious to delicate invalids during the winter and spring, are the north-west winds which are generally dry, sharp, and cold. Compared, however, with the climate of the coast of America, under the same latitude, Bermuda may be said to have no winter. The summer is very hot, being generally admitted, I believe, by those who have experienced both climates to be more oppressive than the same season in the West Indies. This may be accounted for, partly from the want of the trade winds, and partly from the bare, arid nature of the soil, which becomes quite parched during the summer. Vegetation almost disappears at this season; the cedar and wild sage alone resisting the heat. Dew is occasionally deposited in winter, when a cold night succeeds a hot day, but never in the summer."

Lying in the same parallel of latitude with Madeira, the Bermudas are far inferior to the former in point of climate. The Summer temperature of Bermudas is tropical, while that of Madeira is very moderate, owing, no doubt to the influence of the trade winds, and its mountainous character. The Bermudas are not therefore likely to become favourite residences for invalids.

Over the Canaries we may pass, as they possess few, if any advantages over Madeira, and are far inferior in many respects. The Azores possess a climate, we believe, superior even to that of Madeira; but the want of accommodations and of medical advice must, for a long time to come, render the fineness of the climate nugatory.

### III. WEST INDIES.

Of late years a good number of invalids have resorted to the West Indies to escape the English Winter. It becomes therefore some object to investigate the climate of these islands. Dr. C. confines his observations to the windward or Caribean Islands, which are those most likely to be frequented by invalids. There is considerable difference in the salubrity of these isles, and in the different localities of the same island. Generally speaking the high islands are the more healthy—the low and marshy insalutary. Unfortunately most of the harbours in the West Indies are in the immediate vicinity of swamps or marshes, the towns being built more with reference to commerce than health. The islands which Dr. C. proposes to notice are, Barbadoes, St. Vincent, St. Kitts, and Antigua. These possess advantages for the invalid which render them preferable to all other parts of the Caribean Isles. St. Vincent and St. Kitts are mountainous—Barbadoes and Antigua low; but as St. Vincent lies in the neighbourhood of Barbadoes, and St. Kitts near Antigua, the invalid has an opportunity of changing from low to high ground, and vice versa, as occasion may require.

"The mean annual temperature of the West India Islands, near the sea, is about 79° or 80°. The mean daily range is only about 6°; and the extreme annual range is not more than 20°. The mean temperature of the sea, at considerable depths in the vicinity of these islands, is 80°; and this is also the temperature of the springs near the level of the sea in Jamaica, as noticed by Dr. Hunter. The mean temperature of some of the habitable spots of the mountain ranges is probably not more than 65 or 70°.* The mean temperature of

---

* On the summit of the blue mountain peak, the highest land in Jamaica, 7,555 feet above the level of the sea, the thermometer was found to range from the seasons, according to the European division adopted in this work, is at Barbadoes as follows,—Winter, 76°7; Spring, 79°; Summer, 81°; Autumn, 80°.

"The above applies to the whole of these islands near the level of the sea; the difference in different islands being scarcely worth remarking. The mean temperature of Barbadoes, according to Hillary, is 79°3; the greatest range in six years being 17°, viz. from 70° to 87°: and Dr. Thomas makes this only 18°, from 70° to 88°. Sir Gilbert Blane once found the thermometer in this island at sun-rise, in December, at 69°. Dr. Hunter observed it once only at the same degree; and twice only as high as 91° in Jamaica. The greatest range which I find noticed by any author at the sea level is 22°, viz. from 70° to 92°. Dr. Fergusson says, the mean daily range in summer is from 80° to 86°, and in winter from 70° to 80°. The mean temperature of Grenada, at noon, according to Dr. Chisholm, is 84°, and at seven a. m. 78°5. This gentleman gives the following, as the diurnal progression of temperature. 'The Thermometer (Fahrenheit's) almost universally exhibits the following movements. At seven a. m. the mercury begins to rise, and continues to do so till one p. m., from which time to four p. m. it is stationary. It then begins to fall, and continues to do so till ten p. m., from which time till seven a. m. it is again stationary. This routine of temperature is disturbed only when any remarkable change takes place in the atmosphere, such as, much rain attended with strong wind: the greatest change from this cause I have observed is 10°, the least 4°. The thermometer, exposed to the direct rays of the sun, has risen in ten minutes to 130°, or 42° above its stationary point at one p. m. of that day; 30° may, however, be considered the medium difference between the heat of the shade and in the sun. The medium difference between the heat of the atmosphere at one and ten p. m. is 9°.'" 17.

The Winter and early part of the Spring is, in general, remarkably dry, and the weather fine, the wind being more northerly than usual. The Summer is dry and hot—the Autumn affected with heavy rains. There is but little of that continuous rain which we see in Europe. A medical friend informed our author that he had repeatedly examined, in every season, and at various hours of the night, the grass and bushes of the Caribean Islands, but never found them even damp, from the slightest precipitation of dew. This remarkable fact seems accounted for by the small diurnal range of temperature in these Islands.

"From the small size of the greater number of these islands, there does not occur the regular alternations of land and sea breezes which prevail generally in tropical climates, but the same circumstance admits of the constant influence of the easterly, or trade wind, without intermission. This wind prevails, with great regularity, for nine months of the year. During August, September, and October, the season of rain and hurricanes, the trade winds are much more irregular, but still the prevailing wind is decidedly the East. It is chiefly owing to the full influence of this wind that the climate of the West India Islands is tolerable, and that the temperature of the air at sea is so uniform."

"From this account of its temperature alone, there is no difficulty in drawing the conclusion, that the climate of the West Indies is an improper one, generally speaking, for consumptive patients. It is too hot during the night; and during the day, the high temperature and cloudless skies almost entirely defeat one of the chief objects for the attainment of which the invalid migrates to a warmer climate; I mean exercise in the open air. He could scarcely venture

---

47° at sun-rise, to 58° at noon, in August.—*Observations on Diseases of the Army in Jamaica, by* John Hunter, M. D., &c.

to take exercise, even on horseback, after seven o'clock in the morning; during the coolest season; and, as there is hardly any twilight within the tropics, he would not be able to enjoy the coolness of the evening, in this way. If we have found cause to condemn Italy as a summer residence for consumptive patients, there seems no just reason why we should commend the West Indies, even in winter, the temperature of which is above the summer temperature of any place in the south of Europe. If to this consideration we add the numerous privations, annoyances, and discomforts which are almost inseparable from a residence in the West Indies, I think we might almost be justified in erasing these islands from the list of places suited to the phthisical invalid. Among other contingent disadvantages may be mentioned the difficulty of procuring houses in proper situations, the expences of living, the annoyance of musquitoes, sandflies, &c. &c.

"If to these objections, founded on an impartial consideration of the nature of the climate and of the disease, we add those of a more conclusive nature, derived from the experience of medical men, I conceive the question of the propriety of sending patients labouring under confirmed consumption to the West Indies, will be set at rest for ever.

"In the first place, I may remark, that tubercular phthisis is by no means rare, even among the white inhabitants of the West India islands, while it is of frequent occurrence among the black; and it is not uncommon for individuals affected with this disease to migrate in search of health to a more northern climate.

"These circumstances, however, although properly noticed here, are not adduced as arguments against the propriety of sending consumptive patients to the West Indies; because we find phthisis prevailing, in a greater or less degree, among the natives of every civilized country in the world. But a very different conclusion must be drawn from the fact confirmed to me by numerous medical friends, who have resided in the West Indies—that consumptive cases sent thither proceed much more rapidly to a fatal termination than in temperate climates. And, indeed, this is what we should expect, *a priori*, from considering the nature of the disease, and the well-known influence of the summer climate of the south of Europe on its progress. I am not, however, prepared to maintain that cases of consumption do not occasionally present themselves, in which, even in the advanced stages, a temporary residence in the West Indies might not prove useful. But I do venture to affirm, that such instances are of comparatively rare occurrence, and I would scarcely attempt to designate them. If there are any such, they will be found among the more chronic examples of the disease, which occur about the middle period of life, and which are attended with little constitutional excitement. But I advance this merely as a suggestion, founded on what I know of the disease, as occurring in certain constitutions, and the effects of climate upon these, rather than from practical experience of the effects of that of the West Indies. More extended experience, and more accurate observation than has hitherto been applied to pulmonary invalids sent abroad, can alone enable us to speak positively on this point. In the mean time, every thing that we know regarding the nature of consumption, and the influence of a high temperature on it—supported by our practical experience of the effects of the climate now under consideration, bear us out in laying it down as a general rule—that the climate of the West Indies is an improper one for consumptive patients." 24.

In respect to those people who are only predisposed to phthisis, or what is called threatened with that disease, there is some diversity of opinion among resident practitioners in the West Indies. The subject indeed is a difficult one to investigate. Dr. Ferguson is in favour of the climate as a prophylactic; but much will depend on the nature of the individual's constitution, viz. whether it is calculated to bear the heat of a tropical latitude, or likely to sink under the irritating and exhausting effects of heat.

"When the morbid condition of the system, which gives reason to fear the approach of phthisis, depends chiefly upon hereditary predisposition, and occurs in early life, especially in feeble, irritable constitutions, the climate of the West Indies will disagree. When it occurs at a more advanced period of life, and in a constitution free from much disorder of the nervous system, and of the digestive organs, a temporary residence there may prove useful. The revolution effected in the distribution of the circulating fluids and in the secretions, may have the effect of enabling a constitution in which there exists considerable powers, to overcome the tuberculous diathesis." 26.

The inconvenience of the voyage, especially to females, and the want of accommodations and comforts in a foreign and tropical climate, are circumstances which ought also to be well weighed before undertaking such an important step. In fine, we may safely conclude that the cases of consumption in which the climate of the West Indies promises advantage are very few, and their characters scarcely ascertained—if ascertainable; while those in which it produces mischievous effects are numerous, and generally well-marked. "Even of persons predisposed to the disease, the proportion can be but small who are likely to be benefited by the climate." Chronic diseases of the bronchial membrane are those most likely to be ameliorated by residence in the West Indies, especially if the constitution be otherwise tolerably sound. This is the opinion of Dr. M'Arthur, the talented physician of Deal, who resided several years in Antilles. He considers the climate of the West Indies, however, as unfavourable to asthma. In stomach complaints there can be no doubt that a tropical climate, east or west, is injurious. Chronic rheumatism has been supposed, more from theory than observation, to be one of those maladies for which a tropical climate must offer advantages. The authority above-mentioned, Dr. M'Arthur, considers the climate of the West Indies as favourable to those cases where the constitution is otherwise sound; "but when the health is deteriorated (and it is seldom otherwise) the powers of the digestive organs weakened, or the disease attended with profuse perspirations, nothing but a return to a cooler climate can save the patient." Nothing, indeed, is a more common cause for invaliding.

In scrofula affecting the external parts of the body, the West India climate has been considered as favourable, especially by Dr. Ferguson.

Of all the West India islands, Barbadoes is probably the best for an invalid, being cultivated throughout, free from marshes, level for exercise, and furnished with many accommodations. The capital, however, (Bridgetown) is not very salutary—the most salubrious part of the island is a place called Scotland, 800 feet above the level of the sea, and for ever fanned by the trade winds. It is remarkable that change of air, even from one healthy island to another, has always been observed to be highly beneficial—and this is one cause of the salutary effects of travelling. Kingston, the capital of St. Vincent, has the singular advantage of being built in a salubrious situation on the shores of a fine bay. It is therefore a healthy residence. A cooler station may be found by ascending the mountains; but no accommodations are there to be expected.

The reader will perceive, from this short article, that considerable and valuable additions have been made to this new edition of a work which will become a classical standard of reference on the subject, for the profession in this country.

